# Determinants of Frequent Attendance in Primary Care. A Systematic Review of Longitudinal Studies

**DOI:** 10.3389/fmed.2021.595674

**Published:** 2021-02-09

**Authors:** André Hajek, Benedikt Kretzler, Hans-Helmut König

**Affiliations:** Department of Health Economics and Health Services Research, University Medical Center Hamburg-Eppendorf, Hamburg, Germany

**Keywords:** frequent attendance, high utilization, heavy users, primary care, GP, general practitioner, systematic review

## Abstract

**Introduction:** There is a lack of a systematic review synthesizing longitudinal studies investigating the determinants of frequent attendance in primary care. The goal of our systematic review was to fill this gap in knowledge.

**Methods:** Three electronic databases (Medline, PsycINFO, and CINAHL) were searched. Longitudinal observational studies analyzing the predictors of frequent attendance in primary care were included. Data extraction covered methods, sample characteristics, and main findings. Selection of the studies, extracting the data and evaluation of study quality was performed by two reviewers. In the results section, the determinants of frequent attendance were presented based on the (extended) Andersen model.

**Results:** In total, 11 longitudinal studies have been included in our systematic review. The majority of studies showed that frequent attendance was positively associated with the predisposing characteristics lower age, and unemployment. Moreover, it was mainly not associated with enabling resources. Most of the studies showed that need factors, and in particular worse self-rated health, lower physical functioning and physical illnesses were associated with an increased likelihood of frequent attendance. While most studies were of good quality, several of the included studies did not perform sensitivity analysis or described how they dealt with missing data.

**Discussion:** Our systematic review showed that particularly lower age, unemployment and need factors are associated with the likelihood of becoming a frequent attender. Enabling resources are mainly not associated with the outcome measure. Future research should concentrate on the determinants of persistent frequent attendance due to the high economic burden associated with it.

## Introduction

Primary health care can be defined as an “approach to health and a spectrum of services beyond the traditional health care system. It includes all services that play a part in health, such as income, housing, education, and environment.” Primary care is an “element within primary health care that focuses on health care services, including health promotion, illness and injury prevention, and the diagnosis and treatment of illness and injury” (1). A quite small proportion of patients typically cause a considerable proportion of visits in primary care (2). Consequently, frequent attendance (synonymously, high utilization or heavy use) in primary care is associated with high costs (3). In the same vein, frequent attendance is also associated with future sick leave days and illness-based retirement (disability pensions) (4). Moreover, it should be noted that frequent attendance can cause frustration, stress and burnout among physicians (5–8)—which is important for its own sake and, additionally, these factors can also have an impact on patient and doctor satisfaction (9–13).

The factors associated with frequent attendance in primary care have often been studied based on cross-sectional data [e.g., (14–20)]. An existing systematic review (21) examined the correlates of frequent attendance in European countries. They performed a literature search in November 2016 and mainly identified cross-sectional studies, whereas only one longitudinal study was identified. In total, they particularly found a link between increased need (for example, worse self-rated health or more chronic conditions) and an increased likelihood of being a frequent attender. They also concluded that longitudinal studies are necessary to determine the factors which can contribute to frequent attendance. Actually, in the last years, some longitudinal studies have been published in peer-reviewed journals [for example (22–26)].

More generally, longitudinal studies are needed to improve our understanding of the factors that may contribute to frequent attendance in primary care. Nevertheless, there is a lack of studies systematically synthesizing longitudinal studies which analyzed the determinants of frequent attendance in primary care. Hence, our goal was to provide an overview of the existing longitudinal observational studies investigating the determinants of frequent attendance. Knowledge about the determinants may assist in managing health care use in primary care and might help in decreasing the economic costs linked to heavy or excessive use of primary care (27, 28).

It is worth noting that the determinants of health care use (HCU; including frequent attendance) have often been examined based on the Andersen model of health care utilization (29) which distinguishes between predisposing characteristics like age or sex, enabling resources like income or access to primary care and need factors like self-rated health or chronic illnesses. Moreover, it has recently been proposed to extend the Andersen model by including psychosocial factors like loneliness or personality factors like neuroticism in the Andersen model (30). Therefore, the determinants of frequent attendance are presented based on the Andersen model in our results section.

## Methods

We conducted our systematic review in line with the Preferred Reporting Items for Systematic Reviews and Meta-Analysis guidelines (31). Our systematic review is registered with the International Prospective Register of Systematic Reviews (PROSPERO, registration number: CRD42020178077). Moreover, a study protocol for our systematic review has recently been published (32).

### Search Strategy and Selection Criteria

A systematic literature search was performed in June 2020. To this end, three databases were searched (Medline, PsycINFO, and CINAHL). The search query for Medline is shown in Table 1.

**Table 1:** Search strategy for Medline (systematic literature search performed in June 2020).

#1	Frequent
#2	Use
#3	Consult
#4	Attend
#5	#1 AND (#2 OR #3 OR #4)
#6	Heavy use
#7	High utili
#8	#5 OR #6 OR #7
#9	Physicians, Primary Care
#10	Physicians, Family
#11	Family Practice
#12	General Practitioners
#13	GP
#14	#9 OR #10 OR #11 OR #12 OR #13
#15	Longitudinal
#16	#8 AND #14 AND #15

Using a two-step process (independently conducted by two reviewers, AH and BK), studies were assessed for inclusion/exclusion: (i) Title/abstract screening and (ii) Full-text screening. Moreover, the reference lists of the finally included articles were hand searched (i.e., backwards- and forwards- citation analysis) by both reviewers. In case of disagreements, discussions were used to resolve it (or by including a third party, HHK).

Our inclusion criteria were as follows: (1) Observational longitudinal studies analyzing the factors associated with frequent attendance in primary care in any age group, (2) studies adequately quantifying frequent attendance in primary care, and (3) studies published in peer-reviewed journals in English or German language. Exclusion criteria were: (1) cross-sectional observational studies, (2) studies solely investigating samples with specific disorders, (3) not an observational study, (4) inappropriate measurement of frequent attendance like an uncertain period for frequent attendance, and (5) studies not published in a peer-reviewed journal or in language other than German or English.

We did not apply restrictions with regard to location or time of the studies. Prior to final eligibility criteria, we conducted a pre-test (with a sample of 100 titles/abstracts), though eligibility criteria did not change.

### Data Extraction and Analysis

Data extraction was performed by one reviewer (BK) and a cross-check was performed by a second reviewer (AH). In case of disagreement, discussions were held to reach a consensus (or by inclusion of a third party, HHK). If clarification was required, the study authors were contacted.

We extracted data on study design, assessment of frequent attendance, sample characteristics, sample size, and main findings regarding the determinants of frequent attendance. We present the main findings based on the Andersen model of health care use.

### Quality Assessment

First, it should be emphasized that a consensus on a quality assessment tool for HCU studies does not exist [see also (33)]. Consequently, we used a tool created by Stuhldreher et al. (34) and improved by Hohls et al. (35). This tool was also used in former studies [e.g., (33, 35)]. For further details, please see Table 2. Two reviewers (AH and BK) conducted the quality assessment. If required, discussions were held to reach a consensus or by inclusion of a third party (HHK).

**Table 2:** Quality assessment criteria, adapted from Stuhldreher et al. (34) and Hohls et al. (35).

**Criterion**	**Description**	**x = not fulfilled; ✓ = fulfilled**
**Scope**		
Study objective	Study objective was clearly defined	
Inclusion/exclusion criteria	Clear inclusion and exclusion criteria were given	
**General HCU**		
Frequent attendance description	Frequent attendance was clearly defined	
Comparison group or disorder-specific HCU	The study included a control group without the disorder in order to compare HCU. In case no controls were involved, HCU is associated with disorder of interest, for example, due to the diagnostic code or degree of symptoms	
**Calculation of HCU**		
Data source	The source of information on healthcare utilization was described	
**Study design and analysis**		
Missing data	The proportion of missing data was reported and handling of missing data was reported	
Statistics	The analytical approach was described	
Consideration of confounders	Potential confounding variables were considered in the analyses by adjustment	
Sensitivity analysis	Relevant parameters were varied in sensitivity analysis in order to test the robustness of the results	
**Presentation of results**		
Sample size (subgroup)	The sample size was reported	
Demographics	The characteristics of the sample were described (at least gender and age)	
**Discussion**		
Results discussed with respect to other studies	The results were discussed in relation to comparable studies	
Results discussed regarding generalizability	The results were discussed regarding generalizability to the underlying population	
Limitations	The limitations were discussed	
Conclusion supported by data	The conclusions are supported by the data	
**General**		
Conflict of interest / funders	The conflicts of interest were clearly stated reported for authors and the involvement of funders in the study process was clearly stated	

*HCU, Health care utilization*.

## Results

### Overview of Included Studies

**Figure 1:**
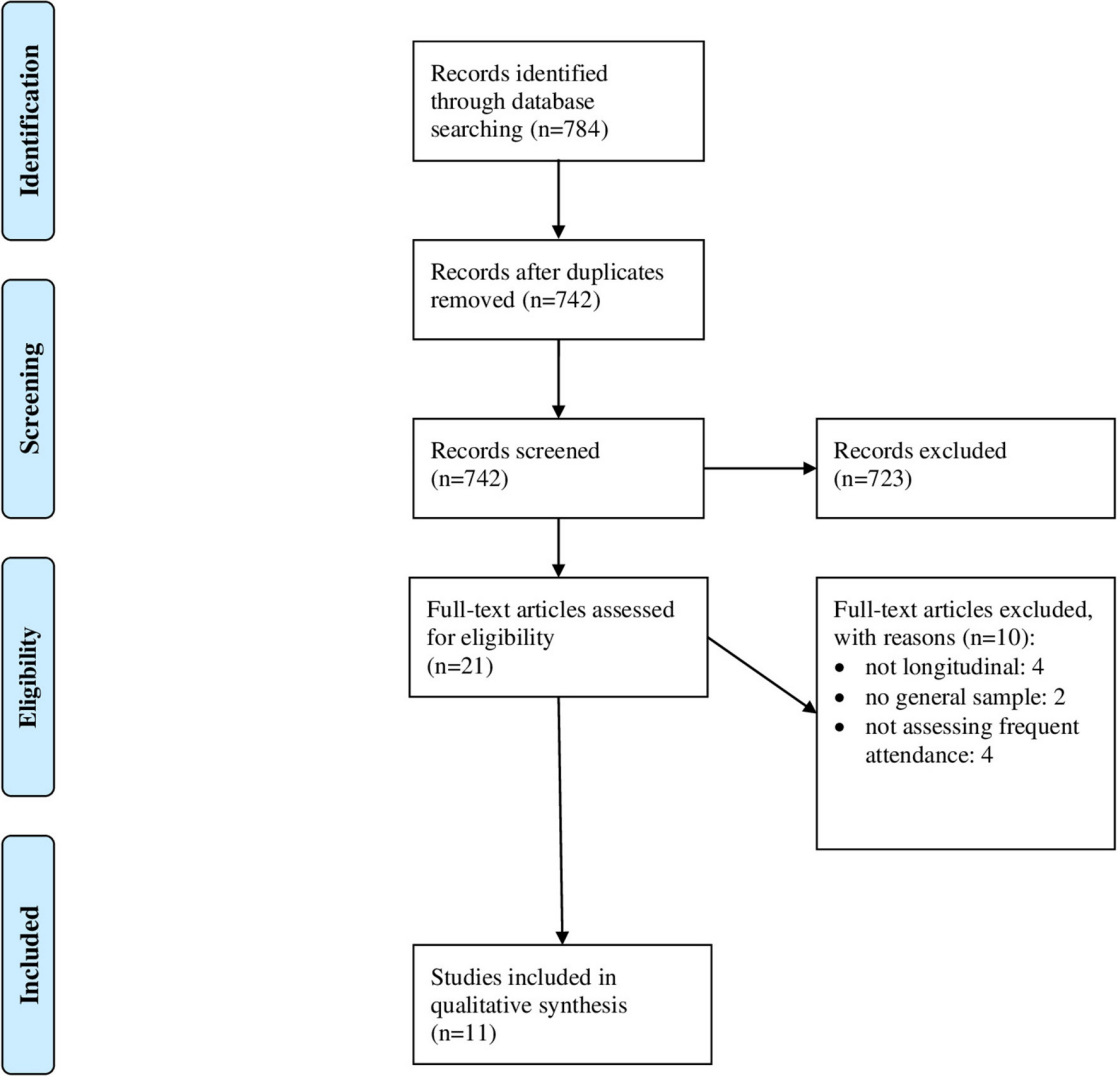
PRISMA flow diagram.

In Figure 1, the study selection process is shown (36). In sum, *n* = 11 studies are included in the final synthesis of our review. In Table 3, key characteristics and main findings of the studies are presented (if reported, adjusted results are presented).

**Table 3:** Key characteristics and main findings of studies included in the final synthesis of the systematic review.

**First author**	**Country**	**Assessment of frequent attendance**	**Sample characteristics**	**Sample size**	**Time span**	**Age**	**Female (%) in total sample**	**Results**
Al-Abadi (37)	Oman	Twelve or more GP visits in the study year	Recruited in primary health care centers	*n* = 12,902	January–December 2008	*M* = 34.0 *SD* = 16.0	61.0%	Comparing median age of frequent and non-frequent attenders, and stratifying for gender showed that frequent attendance was associated with a higher age
Cruwys (38)	United Kingdom	More than one appointment in the last month	Recruited in general practices	*n* = 79	two waves during 4 months	17–37 *M* = 22.0 *SD* = 4.0	67.1%	Regression analysis showed that frequent attendance was significantly associated with being a frequent attender in the previous period (ß = 0.25, *p* < 0.05)
Hadwiger (22)	Germany	Highest decile in terms of GP consultation during the 3 months studied, stratified by sex	German Socio-Economic Panel	*n* = 28,574	seven waves from 2002 to 2014	17–102 *M* = 53.6 *SD* = 16.7	55.6%	Conditional fixed effects logistic regression stated that age (*OR* = 0.95, *p* < 0.001), having a partner (*OR* = 1.23, *p* < 0.01), non-working (*OR* = 1.35, *p* < 0.001), mental health (*OR* = 1.05, *p* < 0.001), physical health (*OR* = 1.12, *p* < 0.001) and non-smoking (*OR* = 1.34, *p* < 0.001) were significantly related to frequent attendance
Hajek (23)	Germany	Six or more GP visits in the study year (= one or more GP visit every 2 month)	German Aging Survey (including community-dwelling individuals in the second half of life, i.e. ≥ 40 years)	*n* = 1,049	Three waves from 2002 to 2011	regarding GP visits: non-frequent attenders (*n* = 541): *M* = 66.9 *SD* = 10.6 frequent attenders (*n* = 508) *M* = 66.3 *SD* = 65.4 regarding specialist visits: non-frequent attenders (*n* = 947) *M* = 64.0 *SD* = 10.9 frequent attenders (*n* = 915) *M* = 64.0 *SD* = 10.9	51.6%	Fixed effects logistic regression revealed that age (*OR* = 0.91, *p* < 0.001), being retired (*OR* = 1.81, *p* < 0.10) or not employed (*OR* = 2.26, *p* < 0.05), the number of physical illnesses (*OR* = 1.18, *p* < 0.01), physical functioning (*OR* = 0.98, *p* < 0.001) and self-rated health (*OR* = 1.40, *p* < 0.001) were significant predictors of frequent attendance at GPs. Frequent attendance at specialists was related to age (*OR* = 0.95, *p* < 0.001), household net income (*OR* = 1.39, *p* < 0.10), the number of physical illnesses (*OR* = 1.24, *p* < 0.001), physical functioning (*OR* = 0.99, *p* < 0.01) and self-rated health (*OR* = 1.50, *p* < 0.001)
Hajek (24)	Germany	Nine or more GP visits in the study year (=highest decile)	German Aging Survey (including community-dwelling individuals in the second half of life, i.e., ≥ 40 years)	*n* = 820	two waves from 2014 to 2017	*M* = 67.6 *SD* = 10.7	54.2%	According to conditional fixed effects logistic regression analysis, frequent attendance is significantly associated with self-perceptions of aging (*OR* = 0.44, *p* < 0.001), age (*OR* = 0.93, *p* < 0.10), self-rated health (*OR* = 1.36, *p* < 0.05) and the total number of physical illnesses (*OR* = 1.12, *p* < 0.10)
Pymont (42)	Australia	Highest decile in terms of GP consultation during the study year, stratified by sex	Personality and Total Health (PATH) through life project	*n* =1,734	Three waves over 8 years	Not specified	Non-frequent attenders: 51.3% occasional frequent attenders: 66.5% persistent frequent attenders: 75.8%	Regarding multiple regression analysis, frequent attendance is significantly associated with being female (*OR* = 1.71, *p* < 0.001), diabetes (*OR* = 3.95, *p* < 0.001), asthma (*OR* = 1.64, *p* < 0.001), thyroid (*OR* = 1.70, *p* < 0.01), arthritis (*OR* = 1.38, *p* < 0.01), depression (*OR* = 1.10, *p* < 0.001), self-reported general health (*OR* = 2.11, *p* < 0.01), accomplishing less (*OR* = 0.72, *p* < 0.01), pain interfere (*OR* = 1.40, *p* < 0.001), not being in the labor force (*OR* = 1.37, *p* < 0.05), financial pressure (*OR* = 1.33, *p* < 0.01), and using medication against blood pressure (OR = 1.73, *p* < 0.001), or against sleep problems (OR = 1.40, *p* < 0.01), or antidepressants (OR = 2.11, *p* < 0.001), or other kinds of medications (OR = 1.79, *p* < 0.001). Additionally, they showed that persistent frequent attendance was associated with gender, depression (baseline), physical conditions, disability and use of medication
Pymont (25)	Australia	Highest decile in terms of GP consultation during the study year, stratified by sex	Personality and Total Health (PATH) through life project	*n* = 1,734	Three waves from 2000 to 2008	“Initially aged in the early 40s”	Not specified	Among men, random effects logistic regressions showed that frequent attendance was associated with diabetes (*OR* = 4.37, *p* < 0.001), asthma (*OR* = 1.69, *p* < 0.05), thyroid (*OR* = 6.74, *p* < 0.01), having any pain (*OR* = 1.83, *p* < 0.01), worrying about one's health (*OR* = 1.59, *p* < 0.05), using antidepressants (*OR* = 2.34, *p* < 0.001), using medications for sleeping (*OR* = 2.11, *p* < 0.01), or using other medications (*OR* = 2.21, *p* < 0.001) For women, frequent attendance was significantly related to diabetes (*OR* = 2.56, *p* < 0.05), asthma (*OR* = 1.78, *p* < 0.05), thyroid (*OR* = 1.57, *p* < 0.01), having any pain (*OR* = 1.89, *p* < 0.01), worrying about one's health (*OR* = 1.72, *p* < 0.01), depression (*OR* = 1.14, *p* < 0.01), being in the highest quartile regarding rumination (*OR* = 0.45, *p* < 0.01), using antidepressants (*OR* = 1.91, *p* < 0.01), medications for sleeping (*OR* = 1.54, *p* < 0.05) and any other medications (*OR* = 1.77, *p* < 0.01)
Pymont (36)	Australia	highest decile in terms of GP consultation during the study year, stratified by sex (= highest decile)	Personality and Total Health (PATH) through life project	*n* = 1,197	Two waves (from 2012 to 2013 on)	Not specified	56.1%	Logistic regression revealed that medium (*OR* = 0.46, *p* < 0.05) or large two payment (*OR* = 0.36, *p* < 0.01), having had some no cost consultations (*OR* = 3.01, *p* < 0.01), diabetes (*OR* = 2.06, *p* < 0.01), epilepsy (*OR* = 7.63, *p* < 0.01), pension (*OR* = 0.42, *p* < 0.01), unemployment (*OR* = 4.00, *p* < 0.05), tertiary education (*OR* = 0.50, *p* < 0.05), and using anxiety or depression medications (*OR* = 1.91, *p* < 0.05) were significantly associated with frequent attendance
Reho (39)	Finland	Highest decile in terms of GP visits in one of the study years	Recruited from an occupational health provider	*n* = 66,831	2014–2016 (data collection during every visit to the occupational health provider)	1 year frequent attenders: age groups: 18–34: 1,661 (25.4%) 35–44: 1,641 (25.1%) 45–54: 1,889 (28.9%) 55–68: 1,337 (20.5%) 2 year frequent attenders: age groups: 18–34: 354 (22.1%) 35–44: 413 (25.8%) 45–54: 473 (29.5%) 55–68: 363 (22.6%) permanent frequent attenders: age groups: 18–34: 128 (21.7%) 35–44: 147 (24.8%) 45–54: 187 (31.6%) 55–68: 130 (22.0%) non-frequent attenders: age groups: 18–34: 19,630 (33.8%) 35–44: 13,648 (23.5%) 45–54: 14,351 (24.7%) 55–68: 10,479 (18.0%)	1 year frequent attenders: 49.9% 2 year frequent attenders: 53.0% permanent frequent attenders: 55.7% non-frequent attenders: 42.8%	Multinomial logistic regression revealed that permanent frequent attendance was significantly associated with all kinds of ICD-10 diseases. The strongest relationships occurred with diseases of the musculoskeletal system and connective tissue (*OR* = 26.85, 95% CI: 18.9–38.2), diseases of the respiratory system (*OR* = 15.55, 95% CI: 11.79–20.52) and systems that were not classified (*OR* = 11.15, 95% CI: 9.36–13.29)
Rifel (40)	Slovenia	Highest decile in terms of GP visits within 1 year	Randomly selected 60 family physicians from the Slovenian national register of family medicine physicians Each family physician recruited 10–20 family practice attendees (from 18 to 75 years)	*n* = 1,118	Baseline 2003 Follow-up: 12–24 months	18–75 *M* = 48.7 *SD* = 14.4	63.4%	Multiple logistic regressions showed frequent attendance at follow up wave 2 was associated with lower physical (*OR* = 0.93, *p* < 0.01) and mental health (*OR* = 0.96, *p* < 0.05). Moreover, it was negatively associated with higher educational level (for example: higher school or university, *OR* = 0.26, *p* < 0.05; reference category: elementary school or less)
Taylor (41)	United Kingdom	Eight or more GP visits in the study year (= highest quartile)	Recruited in general practices	*n* = 410	2 years (two checks in between)	16–82 *M* = 41.6 *SD* = 15.3	71.2%	Logistic regression showed that frequent attendance was significantly related to insecure emotional attachment style (*OR* = 8.46, *p* < 0.01)

Data came from Asia (*n* = 1, Oman), Europe (*n* = 7, with three studies from Germany, two studies from the United Kingdom, one study from Slovenia, and one study from Finland), and three studies from Australia (*n* = 3, all from the country Australia). The period of observation in the included longitudinal studies ranged from 4 months to 12 years.

The majority (*n* = 7) of studies used the highest decile (in terms of frequency of GP visits) as cut-off for being a frequent attender (22, 24–26, 39, 40, 42). Moreover, while two studies used twelve or more GP visits in the study year as cut-off (37, 38), a third study used eight visits as cut-off [= top quartile (41)] and a fourth study used six visits as cut-off (23). While some studies used the self-reported frequency of GP visits [e.g., (22–24)], other studies used administrative data [e.g., (25, 26, 39, 42)]. For example, one study extracted the number of visits from the electronic medical records system in Oman (37).

While most of the studies broadly examined the determinants of temporary or persistent frequent attendance, for instance, one study focused on the link between insecure attachment and frequent attendance (41), another study concentrated on the link between out-of-pocket costs and frequent attendance (26) and a further study concentrated on the link between aging satisfaction and frequent attendance (24). Furthermore, it should be noted that some of the studies included focused on temporary frequent attendance [like (22, 24)], whereas other studies (also) examined persistent frequent attendance [e.g., (25, 40)].

The majority of the studies focused on middle-aged or older individuals. The proportion of women mainly ranged from about 50–70%, with a sample size ranging from 79 to 66,831 individuals. In Table 3, further details are shown. Despite the fact that only three out (22–24) of eleven studies included in our review used the Andersen model as theoretical background, we will present our main findings in the following sections according to the [extended (30)] Andersen model (to increase readability and clarity): predisposing characteristics, enabling resources, need factors and psychosocial factors.

### Predisposing Characteristics

In total, *n* = 7 studies explicitly examined the link between predisposing characteristics (including age, sex and employment status) and frequent attendance. It should be noted that not all seven studies included all of the aforementioned predisposing characteristics.

With regard to age, while one study did not identify a link between age and frequent attendance (40), a second study found a bivariate association between higher age and an increased likelihood of frequent attendance (37). However, studies based on nationally representative samples and using advanced techniques like FE regressions found a robust link between increased age and a lower probability of becoming a frequent attender in Germany (22–24).

With regard to sex, there is inconclusive evidence. For example, while one study found a link between being female and an increased likelihood of being a frequent attender (42), another study mainly did not identify such a link (40). Other studies used sex-stratified regressions [e.g., (25)] or used FE regression techniques [e.g., (22–24)]. In these FE regression models, time-constant factors (i.e., factors that do not vary within individuals over time) like sex can only be included as moderating factors. One study (23) examined whether time-constant factors like sex or educational level moderate the link between need factors (in terms of chronic conditions, physical functioning, self-rated health and depression) and frequent attendance. However, all of the respective interaction terms did not achieve statistical significance. In this context, it may be worth noting that one study found a link between lower educational level and an increased likelihood of becoming a frequent attender (40), whereas the majority of studies included did not investigate this factor.

With regard to employment status, while some single studies did not find an association between unemployment and becoming a frequent attender (24, 40), most of the studies found a link between unemployment and an increased likelihood of becoming a frequent attender (22, 23, 25, 42).

While only one single study (22) found a link between getting married and an increased likelihood of frequent attendance, other studies did not identify such a link (23, 24, 40).

### Enabling Resources

In sum, *n* = 6 studies examined the association between enabling resources and frequent attendance. One study found a link between financial pressure and an increased likelihood of being a frequent attender (42). However, studies relying on more advanced panel techniques consistently did not determine a link between income or financial pressure and the likelihood of becoming a frequent attender (22–25). Moreover, a further study did not find a link between out-of-pocket costs and frequent attendance using a counterfactual model adjusting for selection into cost levels (26).

### Need Factors

In total, *n* = 7 studies examined the association between need factors and frequent attendance. They almost consistently found a link between increased need factors and an increased likelihood of becoming a frequent attender (22–25, 39, 40, 42). More precisely, particularly self-rated health, physical functioning and physical illnesses were quite strongly associated with frequent attendance (22–25, 39, 40, 42).

There is mixed evidence regarding mental health and frequent attendance. For example, while some studies identified a link between mental health, depression, or anxiety and an increased likelihood of becoming a frequent attender (22, 22, 25, 40, 42), other studies did not find a significant link (23–25, 40).

It may be worth noting that two studies identified a link between increased medication use (e.g., for sleep problems or high blood pressure) and an increased likelihood of becoming a frequent attender (25, 42). A further study did not identify an association between cognitive functioning and frequent attendance in primary care (23).

### Psychosocial Factors

In total, *n* = 3 studies examined the link between psychosocial factors and frequent attendance. However, these studies largely differed in the key independent variables. For example, one recent study showed a link between increased self-perceptions of aging and a decreased likelihood of becoming a frequent attender (24). Another study revealed that frequent attendance was significantly related to insecure emotional attachment style (41). Moreover, another study showed that loneliness is not associated with frequent attendance in primary care longitudinally (23).

### Quality Assessment

In Table 4, the quality assessment of studies included in our systematic review is shown. In sum, 81.3–93.8% of the criteria were met by the longitudinal studies included in our review. By far, the categories with the most unmet criteria were how missing data were handled (9% fulfilled) and whether sensitivity analyses were performed (45% fulfilled).

**Table 4:** Quality assessment of studies included in the systematic review.

**First** ** author** ** (year)**	**Study** ** objective**	**Inclusion** ** and exclusion** ** criteria**	**Frequent** ** attendance** ** description**	**Comparison** ** group or** ** disorder-specific HCU**	**Data** ** source**	**Missing** ** data**	**Statistics**	**Consideration of** ** confounders**	**Sensitivity** ** analysis**	**Sample size** ** (subgroup)**	**Demographics**	**Results discussed** ** with respect** ** to other studies**	**Results discussed** ** regarding generalizability**	**Limitations**	**Conclusion** ** supported** ** by data**	**Conflict of** ** interest/funders**	**% of criteria** ** fulfilled by** ** study**
Al-Abadi (37)	✓	✓	✓	✓	✓	x	✓	✓	x	✓	✓	✓	✓	✓	✓	✓	87.5
Cruwys (38)	✓	✓	✓	✓	✓	x	✓	✓	x	✓	✓	✓	x	✓	✓	✓	81.3
Hadwiger (22)	✓	✓	✓	✓	✓	x	✓	✓	✓	✓	✓	✓	✓	✓	✓	✓	93.8
Hajek (23)	✓	✓	✓	✓	✓	x	✓	✓	✓	✓	✓	✓	✓	✓	✓	✓	93.8
Hajek (24)	✓	✓	✓	✓	✓	x	✓	✓	✓	✓	✓	✓	✓	✓	✓	✓	93.8
Pymont (42)	✓	✓	✓	✓	✓	✓	✓	✓	x	✓	x	✓	✓	✓	✓	✓	87.5
Pymont (25)	✓	✓	✓	✓	✓	x	✓	✓	✓	✓	x	✓	✓	✓	✓	✓	87.5
Pymont (26)	✓	✓	✓	✓	✓	x	✓	✓	✓	✓	✓	✓	✓	✓	✓	✓	93.8
Reho (39)	✓	✓	✓	✓	✓	x	✓	x	x	✓	✓	✓	✓	✓	✓	✓	81.3
Rifel (40)	✓	✓	✓	✓	✓	x	✓	✓	x	✓	✓	✓	✓	✓	✓	✓	87.5
Taylor (41)	✓	✓	✓	✓	✓	x	✓	✓	x	✓	✓	✓	✓	✓	✓	✓	87.5
% of criteria fulfilled by studies	100	100	100	100	100	9	100	91	45	100	82	100	91	100	100	100	

*x = not fulfilled; ✓ = fulfilled*.

## Discussion

The aim of this systematic review was to provide an overview of the existing longitudinal observational studies investigating the determinants of frequent attendance.

With regard to *predisposing characteristics*, only lower age and (to a lesser degree) unemployment were almost consistently associated with a higher likelihood of becoming a frequent attender, whereas there is rather inconclusive evidence regarding sex, educational level and marital status. At first glance, the link between age and frequent attendance may appear counterintuitive. However, a possible link may be that with increasing age individuals become less optimistic about the treatment or may have increased perceived opportunity costs with regard to physician visits (23). Moreover, the link between a job loss and frequent attendance may be driven by changes in health behavior associated with unemployment (43). In conclusion, only some predisposing characteristics were consistently associated with frequent attendance, whereas there is inconclusive evidence regarding several predisposing characteristics. Future longitudinal studies are required to shed more light on these factors.

With regard to *enabling resources*, existing studies almost consistently did not determine an association between enabling resources (like income) and frequent attendance. These findings may be mainly driven by the characteristics of the health insurance systems of the included studies. Thus, future research, particularly from countries where the ownership of the healthcare system is mainly in private hands (e.g., United States) is required because enabling resources commonly play a key role in these countries. Furthermore, future studies are necessary to examine the link between (perceived) access to primary care and frequent attendance.

With regard to *need factors*, most of the included longitudinal studies found an association between need factors (particularly physical functioning, and physical illnesses) and frequent attendance (both, temporary and persistent). This is highly plausible and in line with various cross-sectional studies (21). However, it should be noted that various studies did not show a link between depression and frequent attendance. This may be explained by the fact that individuals with depression are often referred to the specialist. In total, these findings may indicate that patients mainly have frequent primary care visits when medically indicated. More research is required to examine the link between specific diseases or disease clusters and frequent attendance longitudinally. Furthermore, longitudinal studies focusing on the link between functional complaints (i.e., medically unexplained symptoms) and frequent attendance in primary care are required (41, 44). For example, it has been shown that frequent attenders with medically unexplained symptoms have an increased use and increased costs of medical investigations (45).

With regard to *psychosocial factors*, only some single studies exist which largely differed in their key independent variables. Therefore, we refrained from drawing conclusions from these single studies. It should be emphasized and repeated that while most of the included studies focused on rather conventional explanatory variables like sex, age or health-related factors, psychosocial factors like loneliness or satisfaction have rarely been examined. Since, for example, a recent cross-sectional study has demonstrated that after adjusting for various covariates, psychosocial factors are still important determinants of frequent attendance (16), we hope that future longitudinal studies close this gap in knowledge. Moreover, it has been demonstrated that personality factors like neuroticism are important for health care use (33, 46, 47). Furthermore, personality-related factors like external health locus of control (i.e., the belief that health depends on others like GPs) may heavily drive frequent attendance in primary care (47). Moreover, factors such as increased health anxiety may be of importance for an increased likelihood of being a frequent attender (48, 49). Therefore, we also hope that future research will shed light onto the link between personality factors (in a broader sense) and frequent attendance.

Against this backdrop, the importance of the conventional Andersen model as a theoretical foundation for analyzing the determinants of frequent attendance in primary care can be critically discussed. However, we think that this extended version (including psychosocial and personality-related factors) offers a promising theoretical framework in this context (30).

The variety in study quality between the studies was rather low and, in general, the study quality of the included studies was rather high. The general high quality is rather unsurprising given the fact that all studies have been published in the last 10 years. However, common shortcomings of the included studies are that more than one half of studies included did not perform sensitivity analysis. This is, however, of importance to verify the robustness of the study findings and current guidelines therefore recommend these robustness checks (50). Moreover, only one study described how missing data were handled. This can result in, among other things, biased parameter estimates, biased standard error estimates or a severe loss of statistical power (51). Future studies should overcome this limitation [e.g., by using techniques such as full-information maximum likelihood (52)] because these missing-data techniques may result in more accurate and reliable results (51). Moreover, only three studies used the Andersen model as theoretical background and for selection of independent variables. However, in total, we cautiously assume that these shortcomings (i.e., how missing data were handled and absence of sensitivity analysis) did not heavily affect the robustness of our review's findings. Nevertheless, we cannot dismiss the possibility of a publication bias. Moreover, future research is required to clarify how exactly these shortcomings regarding missing data and sensitivity analysis can affect the robustness of systematic reviews.

In total, and related to the quality of the studies, there are some factors that restrict the comparability of the studies included in our review. While some studies are based on individuals recruited in general practices, other studies used data from region-wide (25, 26, 42) or nationally representative (22–24) samples. Moreover, one study used a random and representative sample of Slovenian family medicine practices' attenders (40). While most of the studies used the highest decile to define frequent attenders, some other studies used cut-offs like at least 12 visits per year (i.e., on average one visit per month). Despite using longitudinal data, only a few studies used regression models specifically designed for longitudinal data (like conditional FE logistic regressions) [for example (22–25)]. However, using these regression techniques is important to deliver consistent estimates (53). Thus, the question remains whether all of the studies included in our systematic review produced consistent estimates and their findings should be therefore interpreted with caution. However, it should be noted that the included studies mostly produced similar results (in terms of direction and significance). Moreover, exclusively focusing on studies using panel regression techniques (with conservative model assumptions) supported our main conclusions (i.e., particularly increased needs are associated with becoming a frequent attender).

Included studies partially used self-reported doctor visits. This, however, may introduce some bias (recall bias) (54). Upcoming research should link survey data with claims data (if data are available) to reduce this potential threat to the validity. As noted above, existing studies focused on a variety of explanatory variables. For instance, while one study focused on aging satisfaction as explanatory variable (24), other studies [e.g., (22, 23)] focused on common explanatory variables based on the Andersen model. Moreover, while some studies focused on temporary frequent attendance, other studies (also) concentrated on persistent frequent attenders [e.g., (40, 42)] as outcome measure. Studies also exist mainly focusing on persistent frequent attenders (44, 55–57). For instance, it has been shown that among 1 year frequent attenders, about one out of six became a persistent frequent attender (44). Furthermore, it should be noted that the existing studies focused on patient characteristics, but not on GP- (including GP–patient relationship) (58, 59) or system-related characteristics (60). These factors may also drive frequent attendance. For instance, a cross-sectional study conducted in Slovenia showed a link between higher satisfaction with the family physician and frequent attendance (61). Moreover, factors such as collusion (acquiescence by doctor to explanation provided by patient) (12, 62) which can contribute to questioning of doctor's openness and competence (12) may ultimately affect the likelihood of frequent attendance. However, further research is required the longitudinal association between GP-/system-related characteristics and frequent attendance in primary care.

Our systematic review also has some strengths and limitations. First, this current work is the first one systematically synthesizing evidence regarding the determinants of frequent attendance in primary care solely concentrating on longitudinal studies. Due to the focus on longitudinal studies, we are quite confident that the studies included have a rather high quality and may provide more valid conclusions regarding factors that can affect frequent attendance. Moreover, a quality assessment was performed. Two reviewers performed main steps like selection of the studies, data extraction and evaluation of study quality. A meta-analysis could not be performed because of the heterogeneity between the studies. Moreover, we restricted our search to peer-reviewed articles. On the one side, this may ascertain a high quality. On the other side, we cannot fully dismiss the possibility that some previous findings (e.g., gray literature or conference abstracts) may be missing. Furthermore, publications in German and English language were included. Again, some studies published in other languages may not be identified in our systematic review. Additionally, our search strategy focused on 16 search procedures. Our search strategy was, among other things, informed by frequently used keywords of relevant articles [such as (25) or (24)]. However, it should be noted that other terms (e.g., related to help seeking) were not included and we restricted our search to studies including the term “longitudinal”. Nevertheless, we assume that our systematic review includes at least most of the studies important to our topic since two reviewers additionally performed a hand search of relevant studies (backwards- and forwards- citation tracking).

## Conclusions

Our systematic review showed that particularly lower age and need factors (thereof, particularly physical functioning and physical illnesses) are associated with the likelihood of becoming a frequent attender. Enabling resources are mainly not associated with the outcome measure. Future research should concentrate on the determinants of persistent frequent attendance due to the high economic burden associated with it. This may assist in mitigating these costs. Moreover, most of the studies included used data from European countries. Future research is required from other regions (e.g., African or Asian countries).

## Author Contributions

The study concept was developed by AH, BK, and H-HK. The manuscript was drafted by AH and critically revised by BK and H-HK. The search strategy was developed by AH and H-HK. Study selection, data extraction, and quality assessment were performed by AH and BK, with H-HK as a third party in case of disagreements. All authors have approved the final version of the manuscript.

## Conflict of Interest

The authors declare that the research was conducted in the absence of any commercial or financial relationships that could be construed as a potential conflict of interest.

## References

[1] Muldoon LK Hogg WE Levitt M. Primary care (PC) and primary health care (PHC). Can J Public Health. (2006)97:409–11. 10.1007/BF03405354 17120883 PMC6976192

[2] van den Bussche H Kaduszkiewicz H Schäfer I Koller D Hansen H Scherer M. Overutilization of ambulatory medical care in the elderly German population?–An empirical study based on national insurance claims data and a review of foreign studies. BMC Health Serv Res. (2016) 16:129. 10.1186/s12913-016-1357-y 27074709 PMC4831189

[3] Von Korff M Ormel J Katon W Lin EH. Disability and depression among high utilizers of health care: a longitudinal analysis. Arch Gen Psychiatry. (1992) 49:91–100. 10.1001/archpsyc.1992.01820020011002 1550468

[4] Reho TT Atkins SA Talola N Sumanen MP Viljamaa M Uitti J. Frequent attenders at risk of disability pension: a longitudinal study combining routine and register data. Scand J Public Health Suppl. (2020) 48:181–9. 10.1177/1403494819838663 30973068 PMC7042497

[5] Neal R Heywood PL Morley S Clayden A Dowell A. Frequency of patients' consulting in general practice and workload generated by frequent attenders: comparisons between practices. Br J Gen Pract. (1998) 48:895–8. 9604412 PMC1409909

[6] O'dowd T Five years of heartsink patients in general practice. Br Med J. (1988) 297:528–30. 10.1136/bmj.297.6647.528 3139188 PMC1840368

[7] Stone L. Blame, shame and hopelessness: medically unexplained symptoms and the'heartsink'experience. Aust Fam Phys. (2014) 43:191. 24701621

[8] Vedsted P Christensen MB. Frequent attenders in general practice care: a literature review with special reference to methodological considerations. Public Health. (2005) 119:118–37. 10.1016/j.puhe.2004.03.007 15694959

[9] Jackson JL Kroenke K. Difficult patient encounters in the ambulatory clinic: clinical predictors and outcomes. Arch Intern Med. (1999) 159:1069–75. 10.1001/archinte.159.10.1069 10335683

[10] Linzer M Konrad TR Douglas J McMurray JE Pathman DE Williams ES. Managed care, time pressure, and physician job satisfaction: results from the physician worklife study. J Gen Intern Med. (2000) 15:441–50. 10.1046/j.1525-1497.2000.05239.x 10940129 PMC1495485

[11] McWhinney IR Epstein RM Freeman TR Lingua medica: rethinking somatization. Am Coll Phys. (1997) 126:747–50. 10.7326/0003-4819-126-9-199705010-00037 9139578

[12] Salmon P Peters S Stanley I. Patients' perceptions of medical explanations for somatisation disorders: qualitative analysis. BMJ. (1999) 318:372–6. 10.1136/bmj.318.7180.372 9933202 PMC27727

[13] Wileman L May C Chew-Graham CA. Medically unexplained symptoms and the problem of power in the primary care consultation: a qualitative study. Fam Pract. (2002) 19:178–82. 10.1093/fampra/19.2.178 11906984

[14] Buczak-Stec E Hajek A van den Bussche H Eisele M Wiese B Mamone S. Frequent attendance in primary care in the oldest old: evidence from the AgeCoDe-AgeQualiDe study. Aging Clin Exp Res. (2020) 32:2629–26. 10.1007/s40520-020-01495-2 32108287

[15] Buja A Toffanin R Rigon S Lion C Sandonà P Carraro D. What determines frequent attendance at out-of-hours primary care services? Eur J Public Health. (2015) 25:563–8. 10.1093/eurpub/cku235 25616592

[16] Hajek A Bock J-O König H-H. Association of general psychological factors with frequent attendance in primary care: a population-based cross-sectional observational study. BMC Fam Pract. (2017) 18:48. 10.1186/s12875-017-0621-5 28340559 PMC5366110

[17] Jørgensen JT Andersen JS Tjønneland A Andersen ZJ. Determinants of frequent attendance in Danish general practice: a cohort-based cross-sectional study. BMC Fam Pract. (2016) 17:9. 10.1186/s12875-016-0412-4 26821807 PMC4730631

[18] Kang SC Lin CC Tsai CC Lu YH Huang CF Chen YC. Characteristics of Frequent Attenders Compared with Non-Frequent Attenders in Primary Care: Study of Remote Communities in Taiwan. Multidisciplinary Digital Publishing Institute; Healthcare (2020). 10.3390/healthcare8020096 PMC7349067 32295021

[19] Luppa M Giersdorf J Riedel-Heller S Prütz F Rommel A. Frequent attenders in the German healthcare system: determinants of high utilization of primary care services. Results from the cross-sectional German health interview and examination survey for adults (DEGS). BMC Fam Pract. (2020) 21:1–10. 10.1186/s12875-020-1082-9 31931727 PMC6958724

[20] Rifel J Selic P. Characteristics of elderly frequent attendees in slovene family medicine practices-a cross-sectional study. Mat Soc Med. (2019) 31:93. 10.5455/msm.2019.31.93-98 31452632 PMC6690307

[21] Welzel FD Stein J Hajek A König HH Riedel-Heller SG. Frequent attenders in late life in primary care: a systematic review of European studies. BMC Fam Pract. (2017) 18:104. 10.1186/s12875-017-0700-7 29262771 PMC5738881

[22] Hadwiger M König HH Hajek A. Determinants of frequent attendance of outpatient physicians: a longitudinal analysis using the German socio-economic panel (GSOEP). Int J Environ Res Public Health. (2019) 16:1553. 10.3390/ijerph16091553 31052591 PMC6539949

[23] Hajek A König HH. Which factors lead to frequent attendance in the outpatient sector among individuals in the second half of life? Evidence from a population-based longitudinal study in Germany. BMC *Health Serv Res*. (2018) 18:673. 10.1186/s12913-018-3487-x 30165847 PMC6117977

[24] Hajek A König HH. Self-perceptions of ageing, GP visits and frequent attendance. longitudinal findings from the German ageing survey. Aging Ment Health. (2020). 10.1080/13607863.2020.1742659. [Epub ahead of print]. 32189524

[25] Pymont C Butterworth P. Changing circumstances drive changing attendance: a longitudinal cohort study of time varying predictors of frequent attendance in primary health care. J Psychosom Res. (2015) 79:498–505. 10.1016/j.jpsychores.2015.10.007 26526498

[26] Pymont C McNamee P Butterworth P. Out-of-pocket costs, primary care frequent attendance and sample selection: estimates from a longitudinal cohort design. Health Policy. (2018) 122:652–9. 10.1016/j.healthpol.2018.03.014 29631780

[27] Babitsch B Gohl D Von Lengerke T. Re-revisiting Andersen's behavioral model of health services use: a systematic review of studies from 1998–2011. GMS Psycho Social Med. (2012) 9:Doc11. 10.3205/psm000089 23133505 PMC3488807

[28] Hajek A Brettschneider C Scherer M Stark A Kaduszkiewicz H Weyerer S. Needs and health care costs in old age: a longitudinal perspective: results from the AgeMooDe study. Aging Ment Health. (2020) 24:1763–8. 10.1080/13607863.2019.1673310 31591911

[29] Andersen RM. Revisiting the behavioral model and access to medical care: does it matter? J Health Soc Behav. (1995) 36:1–10. 10.2307/2137284 7738325

[30] Hajek A König HH. Beyond symptoms: why do patients see the doctor? BJGP Open. (2020) 4:bjgpopen20X101088. 10.3399/bjgpopen20X101088 32430301 PMC7330227

[31] Shamseer L Moher D Clarke M Ghersi D Liberati A Petticrew M. Preferred reporting items for systematic review and meta-analysis protocols (PRISMA-P) 2015: elaboration and explanation. BMJ. (2015) 349:g7647. 10.1136/bmj.g7647 25555855

[32] Hajek A Kretzler B König HH. Determinants of frequent attendance in primary care. study protocol for a systematic review of longitudinal studies. Int J Environ Res Public Health. (2020) 17:3710. 10.3390/ijerph17103710 32466103 PMC7277920

[33] Hajek A Kretzler B König HH. Personality, health care use and costs. A systematic review. Healthcare. (2020) 8:329. 10.3390/healthcare8030329 32806553 PMC7551013

[34] Stuhldreher N Konnopka A Wild B Herzog W Zipfel S Löwe B. Cost-of-illness studies and cost-effectiveness analyses in eating disorders: a systematic review. Int J Eat Disord. (2012) 45:476–91. 10.1002/eat.20977 22294558

[35] Hohls JK Koenig HH Raynik YI Hajek A. A systematic review of the association of anxiety with health care utilization and costs in people aged 65 years and older. J Affect Disord. (2018) 232:163–76. 10.1016/j.jad.2018.02.011 29494900

[36] Moher D Liberati A Tetzlaff J Altman DG Group P. Preferred reporting items for systematic reviews and meta-analyses: the PRISMA statement. PLoS MED. (2009) 6:e1000097. 10.1371/journal.pmed.1000097 19621072 PMC2707599

[37] Al-Abadi B Al-Abadi J Al-Fannah W Jeyaseelan L Al-Maniri A Al-Mahrezi A. The prevalence and characteristics of frequent attenders in primary health care in A'Dakhiliyah Governorate of Oman. Oman Med J. (2018) 33:331. 10.5001/omj.2018.60 30038733 PMC6047176

[38] Cruwys T Wakefield JR Sani F Dingle GA Jetten J Social isolation predicts frequent attendance in primary care. Ann Behav Med. (2018) 52:817–29. 10.1093/abm/kax054 30212847

[39] Reho T Atkins S Talola N Sumanen M Viljamaa M Uitti J. Comparing occasional and persistent frequent attenders in occupational health primary care—a longitudinal study. BMC Public Health. (2018) 18:1291. 10.1186/s12889-018-6217-8 30477466 PMC6260555

[40] Rifel J Švab I Selič P Rotar Pavlič D Nazareth I Car J. Association of common mental disorders and quality of life with the frequency of attendance in Slovenian family medicine practices: longitudinal study. PLoS ONE. (2013) 8:e54241. 10.1371/journal.pone.0054241 23342107 PMC3546928

[41] Taylor R Marshall T Mann A Goldberg D. Insecure attachment and frequent attendance in primary care: a longitudinal cohort study of medically unexplained symptom presentations in ten UK general practices. Psychol Med. (2012) 42:855. 10.1017/S0033291711001589 21880165

[42] Pymont C Butterworth P. Longitudinal cohort study describing persistent frequent attenders in Australian primary healthcare. BMJ Open. (2015) 5:e008975. 10.1136/bmjopen-2015-008975 26443661 PMC4606421

[43] Montgomery SM Cook DG Bartley MJ Wadsworth ME Unemployment, cigarette smoking, alcohol consumption and body weight in young British men. Eur J Public Health. (1998) 8:21–7. 10.1093/eurpub/8.1.21

[44] Smits FT Brouwer HJ ter Riet G van Weert HC. Epidemiology of frequent attenders: a 3-year historic cohort study comparing attendance, morbidity and prescriptions of one-year and persistent frequent attenders. BMC Public Health. (2009) 9:36. 10.1186/1471-2458-9-36 19166622 PMC2649070

[45] Reid S Wessely S Crayford T Hotopf M. Frequent attenders with medically unexplained symptoms: service use and costs in secondary care. Br J Psychiatry. (2002) 180:248–53. 10.1192/bjp.180.3.248 11872517

[46] Friedman B Veazie PJ Chapman BP Manning WG Duberstein PR. Is personality associated with health care use by older adults? Milbank Q. (2013) 91:491–527. 10.1111/1468-0009.12024 24028697 PMC3790523

[47] Hajek A König HH. Locus of control and frequency of physician visits: results of a population-based longitudinal study in Germany. Br J Health Psychol. (2017) 22:414–28. 10.1111/bjhp.12236 28345258

[48] Little P Somerville J Williamson I Warner G Moore M Wiles R. Psychosocial, lifestyle, and health status variables in predicting high attendance among adults. Br J Gen Pract. (2001) 51:987–94. 11766871 PMC1314191

[49] Patel S Kai J Atha C Avery A Guo B James M. Clinical characteristics of persistent frequent attenders in primary care: case–control study. Fam Pract. (2015) 32:624–30. 10.1093/fampra/cmv076 26450918 PMC5926457

[50] Von Elm E Altman DG Egger M Pocock SJ Gøtzsche PC Vandenbroucke JP. The Strengthening the Reporting of Observational Studies in Epidemiology (STROBE) statement: guidelines for reporting observational studies. Int J Surg. (2014) 12:1495–9. 10.1016/j.ijsu.2014.07.013 25046131

[51] Allison PD Missing Data. Thousand Oaks, CA: Sage (2001).

[52] Von Hippel PT New confidence intervals and bias comparisons show that maximum likelihood can beat multiple imputation in small samples. Struct Equ Model. (2016) 23:422–37. 10.1080/10705511.2015.1047931

[53] Cameron AC Trivedi PK Microeconometrics: Methods and Applications. New York, NY: Cambridge University Press (2005).

[54] Bhandari A Wagner T. Self-reported utilization of health care services: improving measurement and accuracy. Med Care Res Rev. (2006) 63:217–35. 10.1177/1077558705285298 16595412

[55] Smits FT Brouwer HJ van Weert HC Schene AH ter Riet G. Predictability of persistent frequent attendance: a historic 3-year cohort study. Br J Gen Pract. (2009) 59:e44–50. 10.3399/bjgp09X395120 19192367 PMC2629841

[56] Smits FT Brouwer HJ Zwinderman AH Mohrs J Schene AH van Weert HC. Why do they keep coming back? Psychosocial etiology of persistence of frequent attendance in primary care: a prospective cohort study. J Psychosom Res. (2014) 77:492–503. 10.1016/j.jpsychores.2014.08.003 25217448

[57] Smits FT Brouwer HJ Zwinderman AH van den Akker M van Steenkiste B Mohrs J. Predictability of persistent frequent attendance in primary care: a temporal and geographical validation study. PLoS ONE. (2013) 8:e73125. 10.1371/journal.pone.0073125 24039870 PMC3764153

[58] Heje HN Vedsted P Sokolowski I Olesen F. Patient characteristics associated with differences in patients' evaluation of their general practitioner. BMC Health Serv Res. (2008) 8:178. 10.1186/1472-6963-8-178 18715502 PMC2533311

[59] Potiriadis M Chondros P Gilchrist G Hegarty K Blashki G Gunn JM. How do Australian patients rate their general practitioner? A descriptive study using the General Practice Assessment Questionnaire. Med J Aust. (2008) 189:215–9. 10.5694/j.1326-5377.2008.tb01986.x 18707566

[60] Van Doorslaer E Masseria C Koolman X. Inequalities in access to medical care by income in developed countries. Can Med Assoc J. (2006) 174:177–83. 10.1503/cmaj.050584 16415462 PMC1329455

[61] Kersnik J Scvab I Vegnuti M. Frequent attenders in general practice: quality of life, patient satisfaction, use of medical services and GP characteristics. Scand J Prim Health Care. (2001) 19:174–7. 10.1080/028134301316982405 11697559

[62] Salmon P. Conflict, collusion or collaboration in consultations about medically unexplained symptoms: the need for a curriculum of medical explanation. Patient Educ Couns. (2007) 67:246–54. 10.1016/j.pec.2007.03.008 17428634

